# Challenges for Transformation: A Situational Analysis of Mental Health Care Services in Sehore District, Madhya Pradesh

**DOI:** 10.1007/s10597-015-9893-1

**Published:** 2015-06-10

**Authors:** Rahul Shidhaye, Anusha Raja, Sanjay Shrivastava, Vaibhav Murhar, Rohit Ramaswamy, Vikram Patel

**Affiliations:** Centre for Control of Chronic Conditions, Public Health Foundation of India, 19, Rishi Nagar, Char Imli, Bhopal, Madhya Pradesh India; Maastricht University, Maastricht, The Netherlands; PRIME Project (India), Bhopal, India; Sangath, Goa, India; Public Health Leadership and Maternal and Child Health, University of North Carolina, Chapel Hill, NC USA; London School of Hygiene and Tropical Medicine, London, UK

**Keywords:** Health services research, Mental disorders, Situational analysis, India

## Abstract

The proportion of individuals with mental disorders receiving evidence based treatments in India is very small. In order to address this huge treatment gap, programme for improving mental health care is being implemented in Sehore district of Madhya Pradesh, India. The aim of this study was to complete the situational analysis consisting of two parts; document review of Sehore district mental health programme followed by a qualitative study. The findings suggest that there are major health system challenges in developing and implementing the mental health care plan to be delivered through primary health care system in Sehore district.

## Introduction

Mental disorders constitute around 7.4 % of the global burden of disease (Murray et al. 2012). The disorders significantly contributing to this burden are major depressive disorder, anxiety disorders, drug use disorders, alcohol use disorders and schizophrenia (Murray et al. 2012). In India, the contribution of mental disorders to the overall burden of disease is 11.8 % and this burden is projected to increase during the next 25 years as a consequence of the reduction in burden of communicable diseases and ageing of the population (Patel et al. 2011). At least 1 % of the Indian population, including children, suffers from serious mental disorders (such as psychoses and neurodevelopmental disabilities) and between 5 and 10 % from other types of mental disorders (Reddy and Chandrashekar 1998). Mental disorders are also associated with poor physical health and premature mortality, most notably through suicide. Suicide is now a leading cause of death in young people in India (Patel et al. 2012). Despite this huge burden and severe consequences, it is estimated that only 10 % of those with mental disorders are receiving evidence based interventions (Murthy 2011). The scarcity in availability of mental health services is compounded by huge inequity in access to care as most specialist services are concentrated in urban areas (Murthy 2011).

The National Mental Health Programme (NMHP) was started in 1982 in India with the aim of extending community-based mental healthcare through the existing primary healthcare system (Murthy 2011). The District Mental Health Programme (DMHP) is a flagship mental health intervention programme under NMHP and was conceptualized in mid-eighties (Murthy 2011). The DMHP was implemented in 126 out of 626 districts in India during the eleventh Five Year Plan (FYP) period (2007–2012). The DMHP envisaged a decentralised community based approach to mental health service delivery with emphasis on training of the mental health team, increasing awareness about mental disorders, adequate provision of services to promote early detection and treatment of mental disorders in the community and collecting data for future planning, research and improving service provision. Despite this clear vision, published literature and independent evaluation of the DMHP in India indicate that the programme is, to a large extent, ineffective in practice (Murthy 2011). Some of the reasons for this unsatisfactory state of affairs are: the top-down, ‘one size fits all’ approach to service delivery that cannot accommodate diverse ground realities, poor governance, weak managerial and technical oversight and unrealistic expectations from low-paid, poorly motivated and overburdened primary healthcare personnel (Goel 2011).

In the twelfth FYP (2012–2017), the Ministry of Health and Family Welfare (MOHFW) plans to roll out the DMHP to all districts in a staggered manner and significant financial allocations have been made in the budget outlays. To support this proposed roll-out, the ministry constituted National Mental Health Policy Group in April 2011 to draft India’s first National Mental Health Policy (MOHFW 2012). The huge treatment gap for mental disorders and a strong ‘felt need’ at the national level made a compelling case for designing a Mental Health Care Plan (MHCP) under this policy for mental health services to be delivered through the public health sector in India. The situational analysis of the mental health service delivery described in this paper was carried out in Sehore district of Madhya Pradesh as part of a project to develop these evidence based models. This project, called programme for improving mental health care (PRIME) is a 6 year UKAID supported research programme consortium with a goal to contribute knowledge which informs the development of mental health programs to improve health and socio-economic outcomes in low and middle income country settings (Lund et al. 2012). PRIME is operating in five LMICs (Ethiopia, India, Nepal, South Africa and Uganda) to provide evidence to support the implementation and scale-up of mental health care in primary care and maternal health care settings. In each PRIME country a comprehensive mental health care plan will be developed for ‘districts’ (a geographically defined administrative unit for health service delivery), then implemented, evaluated and scaled-up. A systematic baseline situational analysis in each of the five PRIME study sites was conducted as the first stage of this process to describe the country-specific and cross-country factors relevant to development and implementation of a district-level mental health care plan (Hanlon et al. 2014). In this paper, we present the findings of situational analysis conducted in India which try to broadly address the following key research questions; $1. What is the broader policy environment and overall programme context for implementation of mental health programme in Sehore district? 2. What is the current status of the organization of the mental health services, availability of human and financial resources and to what extent are the mental health services integrated in the primary health care system? 3. What are the gaps in mental health service delivery and what are the reasons for these gaps?

## Methods

### Study Setting

In India, PRIME is implemented through a partnership between Sangath (a Goa based NGO working in the sector of public mental health), the Public Health Foundation of India and the Ministry of Health, Government of Madhya Pradesh. The second largest state in size, Madhya Pradesh (MP) is situated in central part of India and has a population of 72.5 million which accounts for 6 % of the total population of India. As MP has poor general health indicators, it was one of the priority states for UKAID and hence was selected for the PRIME project. Sehore has a population of 1.3 million which is predominantly rural (81 %) and the district covers an area of 6578 km^2^. Sehore district was selected as the administrative health unit for the project because it is one of the districts where the DMHP has already been implemented and therefore the infrastructure to develop, implement and evaluate the Mental Health Care Plan is already in place.

### Study Design

The situational analysis was conducted in two parts. We first completed the document review using a newly developed situational analysis tool (Hanlon et al. 2014) and this was followed by a qualitative study involving interviews with the key stakeholders to broaden and deepen the information obtained from the completion of situational analysis tool.

### Document Review

For the purposes of the PRIME project, a new situation analysis tool (downloadable from http://www.prime.uct.ac.za/index.php/research/tools.html) was developed (Hanlon et al. 2014) as existing methods and tools for appraising mental health systems and services in LMICs did not meet the needs of PRIME either due to reliance on primary research or because they were not applicable to small population units, such as districts and sub-districts (Hanlon et al. 2014). The tool was developed by PRIME cross-country partners with inputs from the Principal Investigators and country team members. This tool tried to capture the information related to broader socio-economic and cultural context, mental health policies and plans, treatment coverage, district level health services, community factors and monitoring and evaluation of mental health services. The details about this tool are published elsewhere (Hanlon et al. 2014).In order to complete the situational analysis tool, state (Madhya Pradesh) and national (India) level documents relevant to general health services delivery such as Project Implementation Plans (PIPs) for state level implementation of National Rural Health Mission, 11th FYP document for state of MP, national and state level human development reports, reports of the National Family Health Survey, reports of the Department of Health Services, Government of Madhya Pradesh, evaluation of the DMHP and other reports related to Sehore DMHP were reviewed. A list of all the documents reviewed is available with the authors and can be shared on request.

The documents mentioned above were largely available in the public domain. These documents were downloaded from the internet and reviewed for information specifically related to mental health services delivery in Sehore. In addition, a formal communication was sent to the DMHP officials in Sehore district and the Psychiatry Department of Government Medical College, Bhopal and to three government officials from the Department of Health Services, Government of Madhya Pradesh to provide any additional documents that may be available. Procurement of the documents, document review and data extraction was completed during August 2011 to October 2011.

The tool was completed by the Project Coordinator and the Intervention Coordinator and was then reviewed by the Principal Investigator for India and PRIME Cross-Country collaborators.

Thus, the situational analysis tool provided the opportunity to identify gaps and areas where further information collection is required. The gaps identified in the document review were used to design a qualitative study to further explore the current status of mental health programme implementation in Sehore district.

### Qualitative Study

The qualitative study consisted of In-Depth Interviews (IDIs) and Focus Group Discussions (FGDs) with the stakeholders and was completed in March–April 2012. Semi structured interview schedules were developed separately for interviews with the policy makers, the health managers and the service providers and FGDs with the community health workers and the members of community covering a range of topics including understanding of the mental disorders, type and quality of the services provided, accessibility and acceptability of the services, adequacy of training and capacity building, and resource constraints and needs.

### Sample

The sampling of respondents for IDIs was purposive to ensure that the perspectives of all the stakeholders in mental health service use and provision are obtained. A total of 11 IDIs were conducted, four with the State level policy makers, three specialists working in the Department of Health Services and four general health service providers and health managers. The state level policy makers included the Principal Secretary of Department of Medical Education, Government of Madhya Pradesh (GoMP), the Principal Secretary of Department of Health and Family Welfare, GoMP, the Health Commissioner, Department of Public Health and Family Welfare, GoMP and the Additional Director-Mental Health, Department of Public Health and Family Welfare, GoMP were interviewed. One psychiatrist who is also the secretary of State Mental Health Authority, GoMP, another psychiatrist who is in-charge of Sehore DMHP and the clinical psychologist involved in Sehore DMHP were also included. The Chief Medical and Health Officer of Sehore district, the District Programme Manager of National Rural Health Mission (NRHM), Sehore and two block level medical officers constituted health manages and service providers for IDIs. Four Focus Group Discussions (FGDs) were conducted in two different blocks i.e. Ashta and Budhni of Sehore district. Out of these, two were with the paramedical staff of PHC (Multi-purpose workers, Pharmacist, Auxiliary Nurse Midwife and Anganwadi workers) and two were with the community members (both male and female members of the village). Eight participants each participated in these four FGDs.

### Data Collection and Analysis

IDIs and FGDs were tape recorded and field notes taken after taking consent from the participants. English interviews were transcribed verbatim while the interviews conducted in the local language (Hindi) were transcribed and translated into English, with back-translation checks performed by the PRIME project team members fluent in the local language (Hindi). A framework analysis approach was used to analyse the qualitative data. An a priori coding framework with a set of high level themes was developed: (1) availability of mental health services, (2) quality of services provided, (3) demand for services focusing on awareness and stigma, (4) rehabilitation facilities, (5) capacity building issues, (6) monitoring and information systems, (7) drug supply, (8) health system requirements for mental health service delivery and (9) mental health policy. Other lower order themes were inductively derived from the data. NVivo9 software (QSR) was used to store and to code the data using the developed coding framework.

## Results

The data from the document review and the qualitative study was triangulated and both service delivery and demand related findings are presented. The service delivery side results are presented using the domains of World Health Organization’s Assessment Instrument for Mental Health Systems (WHO-AIMS). The six domains included in WHO-AIMS are: policy and legislative framework, mental health services, mental health in primary care, human resources, public education, and monitoring and research (WHO 2005). For findings related to the policy and legislative framework domain, the discussion is presented at the national and state levels. The remaining domains are district specific, hence Sehore district level data is presented for these domains.

### Policy and Legislative Framework

#### Mental Health Policy and Plan

At the National level there is no dedicated mental health policy or plan, though the need for same was highlighted by several stakeholders. The National Mental Health Policy Group has submitted a revised plan for DMHP in June 2012(MOHFW 2012). Madhya Pradesh currently does not have the state health policy, mental health policy or mental health care plan at the state level.

#### Mental Health Legislation

National level laws such as the Mental Health Act (MHA) 1987, the Persons with Disabilities Act 1995, and the Rehabilitation Council of India Act 1992 apply to MP and provide a legislative framework to protect people with mental disorders and disabilities in the state. In Feb 2010, the MoHFW initiated the process for drafting a new mental health law to replace the existing MHA 1987. The Mental Health Care Bill has been tabled in the Indian Parliament in August 2013 and it now awaits final approval. As per the recommendations of the MHA 1987, the State Mental Health Authority (SMHA) was formulated in Madhya Pradesh, but its Secretariat is yet to be established and to start functioning regularly.

“Since there is a SMHA, an Additional Director is in a position; even then, the pace is so slow. The programme is visible, but down the lane not much has happened yet.” Senior Government Official; Directorate of Health Services, Madhya Pradesh (India)

#### Mental Health Financing

The state predominately funds the maintenance and up-gradation of mental hospitals. No budget is spent on programs for integrating mental health at the primary care level.

Sehore DMHP is supported by National Mental Health Programme through FYPs. In the 10th FYP (2002–07), the district was allocated Rs 26,20,000 (USD 43,667) in 2003–04. The activities under the DMHP were carried out using these funds till 2011 and the total amount was not fully utilized till then, as a result of which no funds were allocated in 11th FYP. Limited information is available about the expenditure of the allocated funds. PRIME plans to undertake a study to assess the expected resource needs of scaling up services in Sehore district and the state of MP using WHO mhGAP costing tool and compare it with the current financial allocation.

Service providers and officials have expressed that mental health is not a priority for the state, as evident by the negligence in addressing concerns related to mental health programme implementation.

"Mental health is not a priority issue in the State. Therefore, whenever we go and interact with the Chief Medical & Health Officer and Joint Director regarding problems in providing the treatment, they don’t have time to pay attention. Managers feel that other programs need their more attention because they have to complete their targets given by senior officials like family planning and immunizations are most common examples."

DMHP Sehore Staff; Sehore District, Madhya Pradesh (India)

### Mental Health Services: Facility Level

**Fig. 1:**
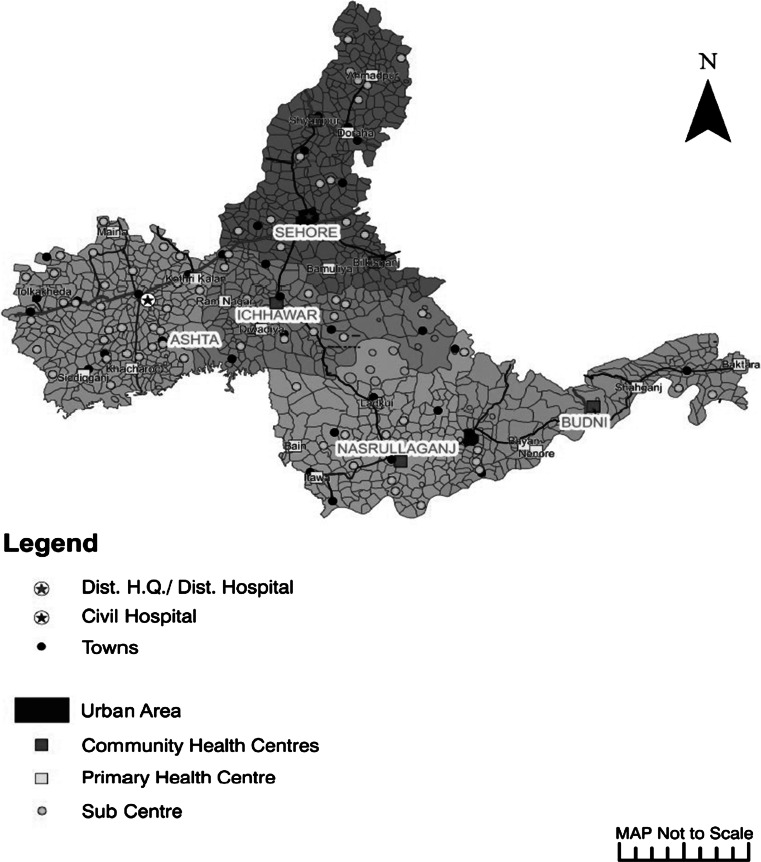
Map of health care facilities in public sector in Sehore district, Madhya Pradesh

The public health system in Sehore district comprises of one district hospital, one urban civil hospital, five community health centers (CHCs), one urban civil dispensary, 17 primary health centers, and 152 sub-health centers. These public health sector facilities are depicted in Fig. 1. The headquarters of the DMHP are in Sehore district hospital.

**Fig. 2:**
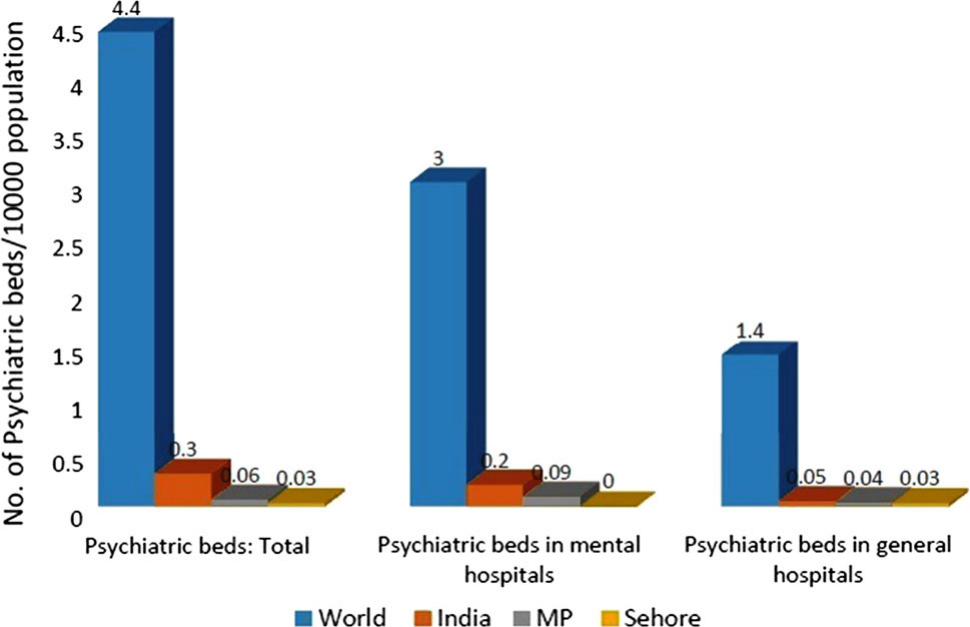
Psychiatric beds in various settings. *Source*: WHO, mental health atlas. Geneva: WHO (2005)

At the state level, in-patient services in the public sector are available at the district hospitals and in the hospitals linked to the government medical colleges. Under Sehore DMHP, patients with manageable acute mental disorders are hospitalized in general male and female wards of the district hospital. No specific facility or wards for mental disorders are present. In addition to this there are two government mental hospitals located at Gwalior and Indore, which provide 379 in-patient beds and utilize 75 % of the funds allotted for mental health through state health budget. National Human Rights Commission reports on both the mental hospitals, state that there is an urgent need to enhance the quality of care with respect to rehabilitative care, nutritional care, and pharmacological and non-pharmacological interventions (Fig. 2).

“Presently, we have provision of four beds to keep mentally ill patients in general medical wards which are always full. We have realized that some time beds are not even available for the mentally ill patients due to increase in the load of patients in the general ward. In general, population or service providers have fear that if mental patients are allowed to treat along with general patients then they may attack other people, because of fear of violence, which we need to address through the information, education, communication.” DMHP Sehore Staff; Sehore, Madhya Pradesh (India)

Out-patient services can be received at district hospitals and government mental hospitals. Sehore DMHP offers free out-patient services conducted by the DMHP psychiatrist and the psychologist on alternate days. Out-patient services are not available at PHCs and CHCs in Sehore district as well as in other districts in MP.

In Sehore DMHP, the number of people treated in the out-patient department (OPD) was significantly higher than the number of people admitted to the hospital. In reported figures from the DMHP Sehore, in 2010, 809 people with major mental disorders attended the OPD and 12 people were admitted. The details about the diagnosis of these patients are not available.

Other services offered by Sehore DMHP include certification of mental disability through intelligence quotient (IQ) and social intelligence (SQ) testing procedures, conduction of mental health awareness camps, and provision of technical guidance to public health facilities and Non-Governmental Organizations working in mental health sector.

The clinical psychologist from the Sehore district hospital provides basic psycho-education and psychosocial support interventions. In the primary health centers (PHCs) and the community health centres (CHCs), no psychosocial interventions are provided. DMHP does not have data on the provision and impact of psychosocial interventions on patients.

No rehabilitation services are provided by any institute for mental disorders. Five alcohol and drug detoxification and rehabilitation centres, supported by the government, are present in MP. In Sehore, there is one private alcohol detoxification centre that is not part of the public health system and is not supported by the DMHP. No specific detoxification ward or facility is in Sehore, but cases are referred to Hamidiya Hospital in Bhopal.

#### Psychotropic Medications

Psychotropic drugs are part of the Essential Drug List, approved by the Department of Health Services, Government of Madhya Pradesh. As mentioned earlier, the DMHP falls within the purview of the Department of Health Services but the Department of Medical Education oversees funding allocation. State officials have expressed challenges in facilitating inter-sectoral coordination when deciding funding distribution, human resource recruitment, and procurement of psychotropic drugs. These medications are not procured by the centralized procurement system because there are no requests generated by the Department of Medical Education, the district hospitals, the CHCs, or the PHCs. This is essentially due to the fact that the staff under the Department of Health Services is not trained in management of mental disorders, as a result of which they neither provide these services, nor do they have any need to put in a procurement request for the psychotropic drugs. Currently; none of the CHCs and the PHCs in Sehore district have thepsychotropic medications available for patients with mental disorders.

### Mental Health Services: Primary Care

The core package of PHC services includes mental health, but it is not implemented. Occasionally, epilepsy services are provided by the PHC doctors and health workers, but no mental health activities or alcohol detoxification related services are provided at the PHC level. No psychotropic medications are available at PHC. Mental health care service provision extends into the community through outreach camps conducted by the psychiatrists and the psychologists. Outreach camps, often half a day long, are organized by the DMHP with support of local community members and with support of NGOs. During the camp general information about mental disorders is provided to the community members. These camps also provide patients the opportunity to meet the psychiatrist and the psychologist for routine check-up and screening for mental disorders. No information about number of individuals attending these camps is available.

### Human Resources

**Fig. 3:**
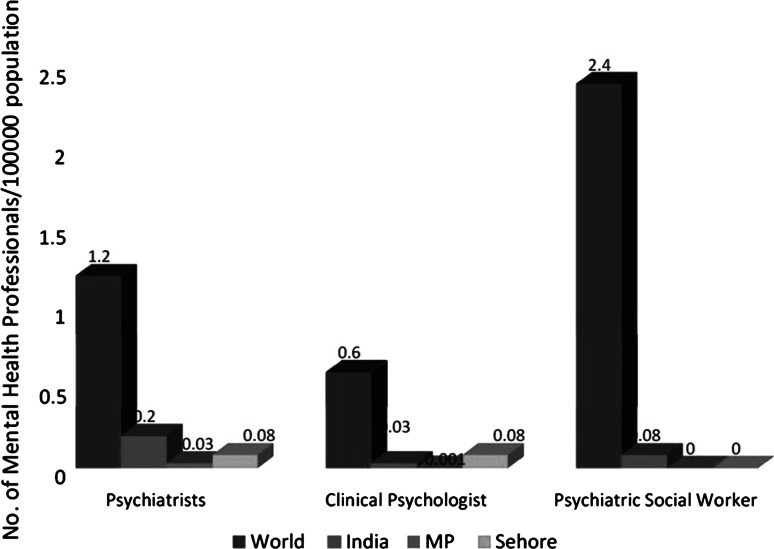
Human resource for mental health services. *Source*: WHO, mental health atlas. Geneva: WHO (2005)

There is severe scarcity of mental health professionals in the state and in Sehore district. There are currently 23 psychiatrist in public sector in MP, just under half of them (11) are employed by the Department of Medical Education (DME) and are based in the Government Medical Colleges and rest (12) are employed by the Department of Health Services (DHS). There is only one clinical psychologist employed in the public sector in the entire state and there is no psychiatric social worker or psychiatric nurse (Fig. 3).

Moreover, psychiatrists are mostly based in urban settings, leading to inequitable access to care. In Sehore, the only psychiatrist and the clinical psychologist in the district are working exclusively for DMHP and providing their services on alternate days.

“In other districts like Dewas, Shivpuri and Mandla, things are not working due to the shortage of human resources. We do not have a psychologist, trained nurses, social workers or psychiatrist who should be located in these districts and likewise in other districts.” DMHP Sehore Staff; Sehore District, Madhya Pradesh (India)

In addition to this, the mental health programme lacks health management staff at the district level. Currently, the Ministry of Health and Family Welfare allots funds to the DME for the coordination of the DMHP. A nodal officer, assigned by DME, is in charge of organizing the DMHP and is the only point person for the program. There is no mental health cadre within the DHS, contributing to the scarcity of mental health professionals and health management staff within the DHS.

“Implementation of the mental health programme should be the responsibility of the Public Health and Family Welfare Department. Medical college should be responsible to organize training programmes. Medical college should play a supportive role while leadership role should be taken by the Public Health and Family Welfare for its implementation.” Senior State Government Official; Department of Medical Education, Government of Madhya Pradesh (India)

### Mental Health Capacity Building

Little effort has been taken to build capacity of PHC and CHC staff by the DMHPs in MP. Only one training was organized by Sehore DMHP team on orientation to mental health for medical officers (MO) and paramedical health workers (HW) based in PHC such as Auxiliary Nurse Midwives (ANMs). According to the district and state officials, health workers such as ANMs and community based paramedical health workers such as Anganwadi workers (AWWs) presently do not have enough knowledge and skills to identify cases from the village and refer to the hospital. Although guidelines and funds are present, getting HWs and MOs in one place for trainings can be difficult. Since many health programs are running in parallel to mental health, trainings have to be organized at a convenient and non-conflicting time and often, mental health is given the least priority.

“All of them are busy in providing health care services in the field and not able to take out time for training. If we call 50 medical officers for training, maximum 20 will turn up. They need self-commitment to carry out the work. All are overworked/overburdened due to shortage of doctors and if more training is provided than they feel more patients would turn up for the treatment at the health facility.” Senior State Government Official; Directorate of Health Services, Madhya Pradesh (India)

“The topic can be integrated into the other training programmes to give importance. As a result, case identification will be easier and functionaries will be able to say that - whether a person is mentally challenged, depressive, psychotic or suffering from alcohol disorder or not.” Senior Official; State Planning Commission, Government of Madhya Pradesh (India)

District and state officials have expressed concern that due to the inadequate capacity building activities, service providers are unable to identify and assess patients’ conditions, hindering treatment provision.

According to the district and state officials, the education system does not support the production of specialists in mental healthcare. Government medical colleges are not offering post-graduate courses on mental health. Mental healthcare is missing from the 12 identified medical subjects in the curriculum.

“There are six government medical colleges but there is not a single seat in post-graduation for mental health. If they would have had at least one seat per medical college then by now we would have six psychiatrists every year. We have the shortcoming of human resources in the State.” Senior Official; State Planning Commission, Government of Madhya Pradesh (India)

### Monitoring and Research

#### Mental Health Information Systems

Documentation and records of inpatient and outpatient cases are maintained at the district hospital. Cases from the district level are reported to the state level based on broad minor, moderate and severe categories and not as per ICD-10 classification of mental disorders. The format does not collect information about specific mental disorder classification for major depressive disorder, alcohol use disorders and psychosis.

“With regard to reporting, since there is no separate space for mental health programmes, so there are no proper reporting procedures. If there would be proper set up, then proper system can be maintained through SMHA. There should be a separate system of reporting and monitoring for mental health programme.” Official of State Mental Health Authority, Madhya Pradesh (India)

There is little to no reporting on current uptake of services taking place at the PHC level due to a lack of monitoring mechanisms. There is a near lack of prevalence and incidence data for the priority mental disorders. No mechanism exists for registering, tracking, and following up mental disorder cases at the PHC level. Moreover, there is a gap in the data regarding the referral mechanism because there is no specific referral mechanism in place from the community to PHC and further up to the district hospital.

### Demand for Mental Health Services

#### Knowledge and Attitudes

The utilization of services is governed by knowledge and attitudes of service providers, users and carers. During FGDs, the community health workers and the community members stated their ignorance about the causes, treatment options, and available mental health services.

“Actually, we do not know about the doctor- who can treat such kind of patients, whether the doctor is capable of treating these patients, sometimes doctor refers a case to another facility, sometimes doctor behave with us very differently.” Community Health Workers; Ashta Block, Sehore District, Madhya Pradesh (India)

Supernatural explanatory models are often used to explain the reason for mental disorders. People rarely reach out to the health services at the PHC and district level; instead, community members turn to traditional and religious healers. Sehore DMHP has not reached out to these alternative resources yet.

“Now many people go to the faith healer for treatment because their parents insist to go to them.” Community Health Workers; Ashta Block, Sehore District, Madhya Pradesh (India)

### Stigma and Discrimination

The prevailing stigma surrounding mental disorders within the community also pose to be a barrier for the utilization of healthcare.

“Currently, villagers cannot identify people who are suffering with mental disorders. The community feels that a person is suffering due to some sinful/bad deeds. They term it as “baadhaa” (obstruction) or “dosh” (fault).” Paramedical Workers; Budhni Block, Sehore District, Madhya Pradesh (India)

The mental disorder ostracizes the person from the community and systems of healthcare available to them. The stigma extends to the family as well. As a result, many people are hesitant to assist a person with mental disorder and abstain from taking the patient to the healthcare setting. The societal discrimination prevents disclosure of mental disorders and familial problems to the community and health workers. Stigma at service-provider’s level is also a major barrier to bringing patients into the health system. Information regarding the treatment gap and stigma and discrimination has not been collected for Sehore district.

“These mental cases are seen as reviled (“grinah”) by the community. If community members try to interfere in the matters of other family members, then family members do not want to hear their views.” Community members; Ashta Block, Sehore District, Madhya Pradesh (India)

### Other Barriers

Community members expressed difficulties in managing a person with mental disorder, who may be uncooperative in the care-seeking process. This issue prevents people from reaching out to services.

“Initially, family members tried to help the alcoholic person to stop drinking. If a person does not listen to the family members then even family members do not care for them. On the other hand, if any patient listens to the family members, they take patient to the private hospital located in Indore for treatment.” Community members; Ashta Block, Sehore District, Madhya Pradesh (India)

The cost of treatment and travel to healthcare centres created another barrier for service utilization. Poor families particularly lack the financial support necessary to avail mental health services.

“Family faces financial crises to take mental patients to the doctors.” Community members; Budhni Block, Sehore District, Madhya Pradesh (India)

## Discussion

The findings of the situational analysis and the qualitative study in Sehore district suggest that there are major health system challenges in developing and implementing mental health care plan that will be able to deliver adequate services for persons with the priority mental disorders through the primary health care system in Sehore district. Severe shortages of skilled mental health professionals in the public health system, very few capacity building initiatives for general health professionals and non-specialists health workers (NSHW) and low mental health literacy and stigma against people with mental disorders has ultimately resulted in non-integration of mental health in primary care in Sehore district.

The low priority given to mental health by policy makers and health planners is a major challenge (Bird et al. 2011). Though Sehore is one of the five districts in MP (out of the total 52 districts) where DMHP is implemented, poor inter-sectoral coordination between various departments responsible for implementation of mental health programs has resulted in under-utilization of allocated funds. The Department of Medical Education which is the grant holding department coordinates poorly with Department of Health Services which is responsible for implementation of public health programs. DMHP funds are released from the Government of India and the process involves multiple bottlenecks. In order to avoid this, the state budget should have dedicated allocation for mental health programs and innovative solutions to leverage the National Rural Health Mission (NRHM) or the local Rogi Kalyan Samit (RKS) funds for procurement of psychotropic drugs or financial support of other programme activities should be sought. Under NRHM, each state prepares a Programme Implementation Plan (PIP) and a log frame based upon the quantum of funds provided to it. These PIPs are consistent with the general principles laid down in the National and State policies relevant to the sector and other agreed action plans. Rogi Kalyan Samiti/Patient Welfare Committee is a registered society which acts as a group of trustees for the hospitals to manage the affairs of the hospital. It consists of members from local Panchayati Raj Institutions (PRIs), NGOs, local elected representatives and officials from Government sector who are responsible for proper functioning and management of the hospital/Community Health Centre/PHCs. RKS is free to prescribe, generate and use the funds with it as per its best judgment for smooth functioning and maintaining the quality of services. Mental Health activities are not yet included in either MP state PIP for NRHM or in the RKS fund allocation.

Scarcity and inequitable distribution of specialist mental health workers is the second major challenge in achieving the goal of improved treatment coverage for mental disorders in Sehore district as well as in MP. Almost complete lack of psychiatric nurses, psychiatric social workers and occupational therapists compounds the problem. There are very few initiatives which have tried to build the capacity of general health staff such as PHC medical officers and non-specialist health workers such as ANMs, Anganwadi workers and ASHAs. It is also imperative to seek innovative solutions through a task-sharing approach emphasizing the delivery of care by NSHW, especially when there is a strong evidence base from South Asian region regarding its effectiveness (Chatterjee et al. 2009; Jordans et al. 2010; Patel et al. 2010; Rahman et al. 2008). NSHW, and the non-formal workforce, such as community volunteers and people with mental disorders and their family members, can be an essential human resource in India; the key questions are to identify their training and supervision needs to deliver evidence based and quality assured treatments. Task-sharing should not be looked as task-replacement. Human resource constraints are accompanied by constraints in other resources such as lack of dedicated in-patient beds for mental health care in District hospitals and inadequate space for out-patient consultations in CHCs and PHCs along with non-availability of psychotropic drugs in these settings.

The third challenge is poor community participation and ownership of the mental health programme. The reduction in the treatment gap depends both on the supply of accessible mental health services as well as on demand for and utilization of mental health services by the community. Stigma towards, and discrimination against, people with mental disorders is an important barrier to mental health service utilization in Sehore.

The fourth challenge is the lack of robust monitoring framework and non-integration of mental health indicators with general health management information systems (HMIS). Mental Health programs should have strong accountability and there should be continuous monitoring and ongoing audit, and periodic evaluation by an independent agency to identify areas of non-performance/reasons so that necessary corrective measures are introduced and evaluated. At present, the data from Sehore district as well as the state of MP for prevalence and incidence of priority mental disorders, help-seeking behavior for these disorders and treatment coverage is practically non-existent. Information on the equitable distribution of treatment coverage, for example, for women, the poor and those living in rural areas, is absent.

One of the limitations of this situational analysis is that it is predominantly based on the documents available in public domain which might have resulted in information bias related to service provision (over-estimation) and service utilization (under-estimation). We tried to further explore the current state of affairs through our qualitative study, but we might not have been able to fully explain the factors which contribute significantly to this situation. However, the findings of this study have provided significant inputs for design of a comprehensive mental health care plan.

Mental Health programs in India are at a critical crossroads, with newly emerging opportunities at the national level which can help radically transform the quality of life of people affected by mental disorders and contribute to reducing the burden of mental disorders and suicide. The MoHFW has plans to roll out the DMHP to all districts in a staggered manner, and it is critical that evidence based models for mental health care delivery are used to inform the design of this roll-out. The situational analysis has served as a first step to recognize these challenges and stresses the importance of addressing supply side constraints (lack of skilled human resources, facilities and psychotropic drugs) and demand side constraints (low mental health literacy and stigma against people with mental disorders) during the development of a comprehensive district mental health care plan for integration of mental health services in primary care.

The output of the PRIME project could be a significant contribution for the roll out of DMHP during the current 5 year plan of the Government of India.

Ethical approval was obtained from Sangath Institutional Review Board before conducting the qualitative research. A participant information sheet containing essential information about the study and the implications of participation were also given to all participants. Participants were requested to sign a consent form to indicate their willingness to participate in the study.
